# Novel Antiviral Activity of Ethyl 3-Hydroxyhexanoate Against Coxsackievirus B Infection

**DOI:** 10.3389/fmicb.2022.875485

**Published:** 2022-04-14

**Authors:** Oluwatayo Israel Olasunkanmi, James Mageto, Juval Avala Ntsigouaye, Ming Yi, Yanru Fei, Yang Chen, Sijia Chen, Weizhen Xu, Lexun Lin, Wenran Zhao, Yan Wang, Zhao-Hua Zhong

**Affiliations:** ^1^Department of Microbiology, Harbin Medical University, Harbin, China; ^2^Department of Cell Biology, Harbin Medical University, Harbin, China

**Keywords:** Coxsackievirus B, ethyl 3-hydroxyhexanoate, antiviral efficacy, natural product, viral diseases

## Abstract

Coxsackievirus group B (CVB) is a member of the genus *Enterovirus* in the family *Picornaviridae*. CVB infection has been implicated as a major etiologic agent of viral myocarditis, dilated cardiomyopathy, meningitis, and pancreatitis among children and young adults. Until date, no antiviral agent has been licensed for the treatment of Coxsackievirus infection. In an effort to identify antiviral agents against diseases caused by the CVB, we found that ethyl 3-hydroxyhexanoate (EHX), a volatile compound present in fruits and food additives, is a potent antiviral compound. In this study, we demonstrated that EHX treatment significantly inhibits CVB replication both *in vivo* and *in vitro*. Furthermore, EHX possesses antiviral activity at 50% effective concentration (EC_50_) of 1.2 μM and 50% cytotoxicity (CC_50_) of 25.6 μM, yielding a selective index (SI) value as high as 20.8. Insights into the mechanism of antiviral activity of EHX showed that it acts at the step of viral RNA replication. Since EHX has received approval as food additives, treatment of CVB-related infections with EHX might be a safe therapeutic option and may be a promising strategy for the development of semi-synthetic antiviral drugs for viral diseases.

## Introduction

Coxsackievirus B (CVB) is a member of the genus *Enterovirus*, the largest genus in the family *Piconaviridae* (Tian et al., 2018). CVB has six serotypes (i.e., CVB1–CVB6). Like other enteroviruses, it has a single-stranded, positive sense RNA of about 7.4 kb (Honkimaa et al., 2020). CVB possesses a long open reading frame (ORF) flanked on both sides by 5′ and 3′ untranslated region (UTR). The ORF is translated into a large monocistronic polyprotein, which is processed into one structural polyprotein (i.e., P1) and two non-structural polyproteins (i.e., P2 and P3) (Huang et al., 2017). P1 is the structural capsid protein (i.e., VP1–VP4) that forms the virus icosahedral capsid structure, while P2 and P3 are the seven viral non-structural proteins (i.e., 2A, 2B, 2C, 3A, 3B, 3C, and 3D) (Tuthill et al., 2010). The non-structural proteins are involved in critical viral genome replication and polyprotein processing. Notably, the viral 3D protein that possesses RNA-dependent RNA polymerase (RdRp) activity is responsible for the viral RNA synthesis (Tuthill et al., 2010). This protein is a potential drug target that aims at the key virus replication step (Wang et al., 2016; Olasunkanmi et al., 2020).

Currently, there are few available drugs licensed as specific treatment for variety of viral infections, and this poses a big concern in the advent of emerging viral infections and mutagenesis of the existing ones. Possible reasons for this setback are proclivity of RNA viruses to develop rapid drug resistance, drug-dependent genetically stable mutations (Strasfeld and Chou, 2010), toxicity issues (Lewis and Dalakas, 1995), and unsatisfactory therapeutic outcomes of potential newly synthesized antiviral compounds. In some cases, drug repurposing and combination have been used as strategies for the treatment of viral infections (Cheng et al., 2019). Although these have shown early success, they pose long-term disadvantages over new drug discovery. All these clearly demonstrate the need to reroute the discovery, design, and development of new antiviral agents. In search for new strategies to develop safe and potent antiviral agents, we proposed that the use of natural products or semi-synthetic parent drugs on natural products could be a promising approach against viral diseases.

Natural products, in particular, plant-derived compounds, remain a valuable source of chemical library that could serve as the chemical variety of options for the development of potent and safe antiviral drugs, which can be used for a range of viral disease (Akram et al., 2018). It forms the basis of treatment of several human diseases and many of the commercially available drugs (Beutler, 2009). Natural products have been a major source of therapeutic or prophylactics agents since ancient times. Typical examples of plant-derived drugs are digoxin and morphine. Worthy of note, in the United States, over 25% of dispensed prescriptions in 1973 were recorded to contain plant-derived active ingredients (Cragg et al., 1997). Until date, bioactive molecules such as essential oils, minerals, flavonoids, esters, and other volatile compounds derived from plants, animals, and microorganisms are still valuable physiological molecules that are used to tackle various metabolic and infectious diseases (Tungmunnithum et al., 2018).

In this study, we reported the antiviral activity of ethyl 3-hydroxyhexanoate (EHX). EHX is a fatty acid ethyl ester of 3-hydroxyhexanoic acid, a key volatile compound in several fruits, such as pineapples, orange juice, cape gooseberry, wood apples, citrus flesh, grapes, tamarillos, and caja fruits (Dunkel et al., 2009; Garg et al., 2018; Medina et al., 2020). The safety evaluations of EHX have received the approval of Joint Food and Agricultural Organization of the United States/World Health Organization Expert Committee on Food Additives (Food and Agriculture Organization of the United Nations [FAOUN], 2002). So far, there is no available report on the antiviral activity of EHX.

Our *in vivo* and *in vitro* data demonstrated for the first time that EHX could inhibit CVB replication by targeting the viral RNA transcription process. Given that EHX is a natural product that has been approved as a food additive, EHX may be a promising agent for the treatment of CVB-related diseases.

## Materials and Methods

### Virus and Compounds

The CVB type 3 Woodruff strain was used for the antiviral experiments. Virus stock made its passage through HeLa cells and was titrated using median tissue culture infectious dose (TCID_50_) assay.

Ethyl 3-hydroxyhexanoate (Aladdin, Shanghai, China) was supplied as 100 mg/100 ml stock solution and was stored at room temperature. Favipiravir (T-705) (Selleck, United States), supplied in powder form, was dissolved in dimethylsulfoxide (DMSO) and was stored at −80°C.

### Mice

Adult Balb/c mice were purchased from the Laboratory of Animal Center of Harbin Medical University (Harbin, China) and were kept in a well-controlled pathogen-free environment at 25 ± 1°C and humidity of 40–50% until they gave birth. Mice were allowed to access food and water *ad libitum*. Care and handling of mice were carried out following the standard procedure as approved by the Ethics Committee of Harbin Medical University, China. Suckling mice (4–5 days after birth) with the average weight of 1.5–2.4 g at administration were infected with 1.5 × 10^6^ TCID_50_ of CVB3 by intraperitoneal injection once at 12 h before treatment.

### Ethyl 3-Hydroxyhexanoate *in vivo* Treatment

Virus-infected mice were administered 250 mg/kg (body weight) of EHX, every 12 h post infection (hpi) (two times per day) by intraperitoneal injection. The total administered volume of the compound was 40 μl. A change in the body weight of each mouse was measured every day and calculated using the formula: (body weight – body weight of the previous day)/5 days. Mice were euthanized at day 5 pi.

### Cell Culture and Virus-Yield-Reduction Assay

All *in vitro* experiments were carried out on monolayer cultures of HeLa cells. The cells were maintained in Dulbecco Modified Eagle Medium (DMEM) supplemented with 10% (v/v) fetal bovine serum (FBS). For virus infection, cells at 60%–70% confluency were infected with the CVB3 at 1 multiplicity of infection (MOI). Following the addition of viruses, cells were allowed to absorb the viruses at 37°C with 5% CO_2_ for 1 h before treatment.

For the virus-yield-reduction assay, HeLa cells were seeded in a 6-well plate and were maintained at 37°C with 5% CO_2_ for 18 h. The cells were infected with virus and were treated with twofold serial dilution of the test compounds. No inhibitor was added to the virus control well. At 24 hpi, virus particles present in the cell culture medium and lysates were collected and quantified using the conventional TCID_50_ assay.

### Cytopathic Inhibition and Cell Viability

To determine the inhibition of CVB-induced cytopathic effect (CPE), HeLa cells were seeded in 6-well plates and were treated with double-fold dilution of EHX. Virus and cell control wells were not treated. CPE of the virus was viewed under the microscope at 24 and 48 hpi. Cell viability was determined using the 3-[4,5-dimethylthiazole-2-yl]-2,5-diphenyltetrazolium bromide (MTT) assay (Meilunbio technology, Suzhou, China) according to the manufacturer’s instructions. The absorbance was read using the SpectraMax microplate reader (Molecular Devices, San Jose, CA) at 542 nm.

Effective and cytotoxicity concentration of the compounds were determined as previously described (Gao et al., 2015). The 50% cytotoxicity (CC_50_) and 50% effective concentration (EC_50_) of each compound were defined as the concentration of the compound that resulted in 50% inhibition of cell viability and the concentration of the inhibitor required to achieve the half-maximal virus inhibition effect, respectively. CC_50_ and EC_50_ were calculated using non-linear regression of GraphPad Prism 6. The selective index (SI) for each compound was calculated as SI = CC_50_/EC_50_.

### Time-of-Addition Assay

To determine the time point at which EHX exerts it antiviral activity against CVB replication, a time-of-addition assay was performed. HeLa cells were seeded in a 6-well plate and maintained at 37°C with 5% CO_2_ for 18 h. Virus-infected cells were treated with EHX (8 μM) at different time intervals as indicated. Total protein was extracted and analyzed by Western blotting at 12 hpi.

### Sodium Dodecyl Sulfate–Polyacrylamide Gel Electrophoresis and Western Blotting

To determine the protein level, total protein was extracted at an indicated time point using radioimmunoprecipitation assay (RIPA) lysis and extraction buffer containing phenylmethylsulfonyl fluoride (PMSF) protease inhibitor (100:1 v/v) on ice. The cells were gently scrapped from the plates and were centrifuged at 12,000 rpm for 15 min at 4°C. The supernatant was collected and stored for further analysis. Protein concentration of the lysates was determined using the bicinchoninic acid (BCA) method. Lysates were separated with 10% or 12% sodium dodecyl sulfate–polyacrylamide gel electrophoresis (SDS-PAGE) and were transferred to polyvinylidene difluoride (PVDF) membrane (Millipore, Billerice, MA). The membranes were blocked with skimmed milk overnight at 4°C and were incubated with primary antibody (Proteintech, United States) at room temperature for 2 h. Blots were further incubated at room temperature with the secondary antibody for 1 h after washing. The anti-3D antibody was raised in rabbits in our lab. Images from blots were viewed with CCD-camera FluroChem M (ProteinSimple, San Jose, CA). Protein intensity was calculated using Image J.

### Quantitative Polymerase Chain Reaction

Total RNA was extracted from the cells using the TRIzol reagent (Invitrogen, Carlsbad, CA). Reverse-transcriptase quantitative polymerase chain reactions (RT-qPCRs) were performed according to the manufacturer’s instruction. Briefly, 1 μg of total RNAs were reverse transcribed in a final volume of 20 μl master mixed reaction: 2 μl of complementary DNA (cDNA), 2 μM of forward and reverse primers, 10 μl of SYBR Premix Ex Taq II, and 6 μl of RNAase-free water were added to make a final reaction volume of 20 μl. The quantitative PCR reaction was carried out for 45 cycles of denaturation at 94°C for 5 s, annealing at 55°C for 15 s, and extension at 72°C for 1 min in LightCycler 96 (Roche, Basel, Switzerland).

### Virtual Screening of the Test Compounds

Putative non-structural and structural proteins of several viruses were retrieved from the Protein Data Bank (PDB). Position constraint was set as follows: distance equals to 2–3 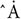 and the energy bonus was −1 kcal/mol. Receptor residue was set at various points. The number of rounds and cutoff were set at 1 and 10, respectively. Favipiravir that is considered as a broad-spectrum antiviral agent was used as a standard control (Joshi et al., 2021). The Mcule drug screening platform^1^ and MedusaDock online software package were used for the virtual screening and docking.

### Statistical Analysis

The mean and standard deviation (SD) of three or more independent experiments are reported. The Student’s *t*-test was used to establish statistical significance (*P* < 0.05) using Graphpad Prism 6 Version 6.02.

## Results

### Ethyl 3-Hydroxyhexanoate Significantly Inhibits CVB3-Induced Cytopathic Effect

**FIGURE 1:**
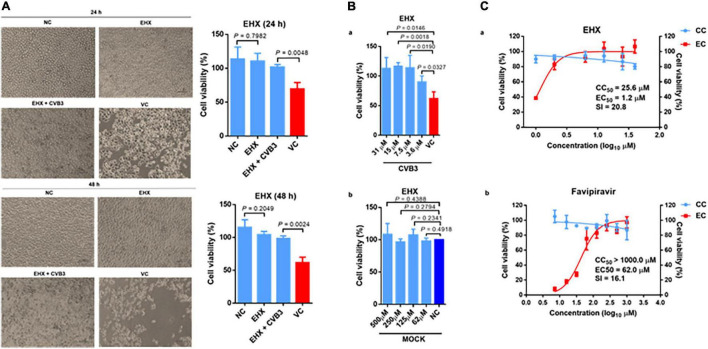
EHX inhibits CVB3-induced cytopathic effect. **(A)** HeLa cells were infected with CVB3 (MOI = 1) and cultured with medium supplemented with EHX (16 μM). The cells were examined microscopically (400×), and cell viability was analyzed at 24 and 48 hpi. **(B) (a)** HeLa cells were infected with CVB3 (MOI = 1) and were treated with 5 μl of the twofold serial dilutions of the compound for 48 h. Cytopathic effect (CPE) was measured by MTT assay. **(b)** HeLa cells were treated with 5 μl of the twofold serial dilutions of EHX for 48 h, and CPE was measured by MTT assay. **(C)** EC_50_ and CC_50_ of EHX and Favipiravir. Twofold serial dilutions of the test compounds were added to virus-infected HeLa cells for 72 h, and the inhibitory effects were analyzed by a CPE assay. Cytotoxicity was examined by incubation of HeLa cells with the indicated concentrations of compounds without the addition of virus. Cell viability was measured by MTT assay and was presented as the percentage of absorbance. CC_50_ and EC_50_ were calculated using non-linear regression (GraphPad Prism 6). Error bars represent SD, *n* = 3, Student’s *t*-test. EHX: ethyl 3-hydroxyhexanoate, VC, virus control; CC, cytotoxicity; EC, effective concentration; SI, selective index; CVB, Coxsackievirus B; NC, negative control.

To gain a preliminary insight into the antiviral activity of EHX, we determined the ability of EHX to inhibit CVB3-induced CPE. To this end, HeLa cells were infected with CVB3 and were cultured in a medium supplemented with EHX (16 μM). Cell viability was determined microscopically and quantified using the MTT assay at 24 and 48 hpi. As shown in Figure 1A, the microscopy and MTT assay revealed that the addition of EHX significantly suppressed CVB3-induced CPE at 24 hpi (*P* = 0.0048) and 48 hpi (*P* = 0.0024) with improved cell viability. Further, another CPE-based antiviral assay was used to measure the antiviral and cytotoxic effect of EHX. This time, varying concentrations of twofold serial dilution of EHX (3.6–30.0 μM and 62–500 μM) were added to virus-infected cells and control wells (i.e., mock test), respectively. The results further demonstrated that EHX significantly inhibits CVB3-induced CPE (Figure 1B), suggesting that EHX may be a potent anti-CVB compound.

Next, we performed a dose-response analysis of EHX. Favipiravir, previously reported as a potent inhibitor of Enterovirus A71 RdRp, was used as a control (Furuta et al., 2017). As shown in Figure 1C, EHX inhibited CVB replication in a dose-dependent manner without significant cytotoxicity to the cell. Notably, the CC_50_ of EHX was estimated to be 25.6 μM, and the EC_50_ was estimated to be 1.2 μM, resulting in an SI value of 20.8 against CVB3. While the CC_50_ of favipiravir was estimated to be >1,000 μM, the EC_50_ was estimated to be 62 μM, resulting in an SI value of 16.1 against CVB3.

### Secondary Confirmation of the Antiviral Activity of Ethyl 3-Hydroxyhexanoate

**FIGURE 2:**
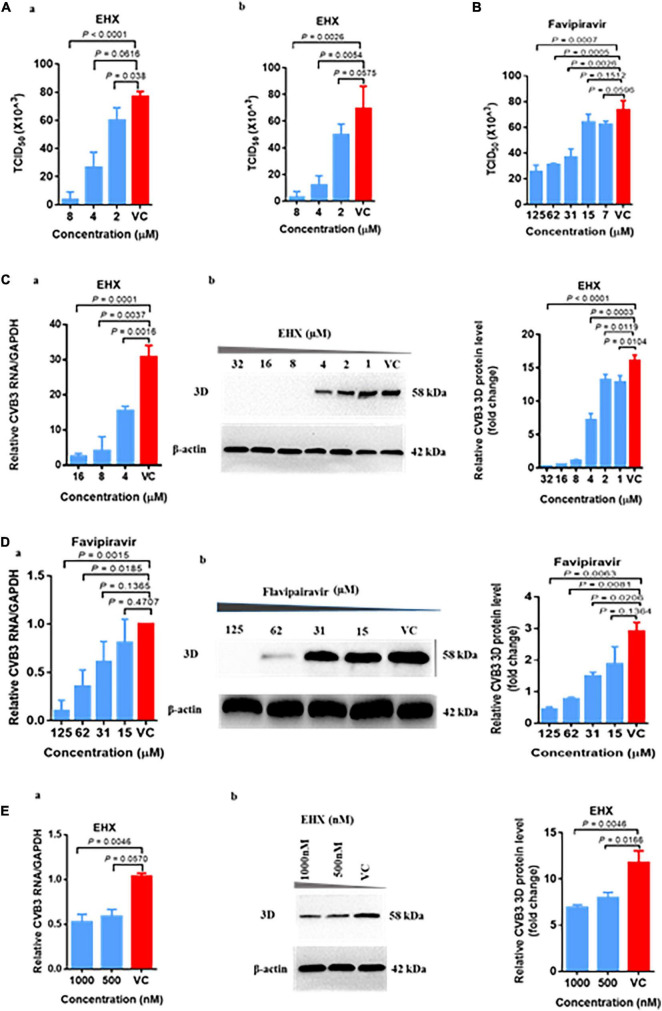
EHX effectively inhibits CVB replication *in vitro*. HeLa cells were infected with CVB3 (MOI = 1) for 24 h. **(A)** EHX inhibits virus-yield. CVB3-infected cells were treated with EHX at the indicated concentrations. **(a)** Cells were subjected to three freeze-thaw cycles; **(b)** culture supernatant were collected and quantified for CVB3 by median tissue culture infectious dose (TCID_50_) assay. **(B)** Favipiravir inhibits virus yield. CVB3-infected cells were treated with favipiravir at the indicated concentrations. Cell culture supernatant were collected and quantified using the TCID_50_ assay. **(C)** EHX inhibits the viral RNA and 3D protein level. CVB3-infected cells were treated with EHX at the indicated concentrations. Total RNAs and 3D protein were analyzed by **(a)** reverse transcriptase polymerase chain reaction (RT-qPCR) and **(b)** Western blotting, respectively. **(D)** Favipiravir inhibits viral RNA and protein level. CVB-infected cells were treated favipiravir at the indicated concentrations. Total RNAs and protein were analyzed by **(a)** RT-qPCR and **(b)** Western blotting, respectively. **(E)** EHX inhibits viral RNA and 3D protein level at a low concentration. CVB-infected cells were treated with EHX at the indicated concentrations. Total RNAs and protein were analyzed by **(a)** RT-qPCR and **(b)** Western blotting, respectively. Error bars represent SD, *n* = 3, Student’s *t*-test.

Next, we performed the virus-yield-reduction assay to validate the antiviral activity of EHX. To this end, CVB-infected HeLa cells were treated with varying concentrations of EHX, and the viruses were quantified using the TCID_50_ assay. As shown in Figure 2A, the results revealed that increasing concentration of EHX (2–8 μM) produced progressive inhibition of CVB replication following infection of HeLa cells with CVB3. In this assay, EHX significantly reduced the titer of CVB3 both in cell lysate at 8 μM (*P* < 0.0001), 4 μM (*P* = 0.0616), and 2 μM (*P* = 0.038) and culture supernatant at 8 μM (*P* = 0.0026), 4 μM (*P* = 0.0054), and 2 μM (*P* = 0.0575) when compared with the virus titer of CVB3-infected cells that were not treated (Figures 2Aa,b). Favipiravir, employed as a positive control, also showed similar anti-CVB properties; however, its dose-response curve was less steep and plateaued at the concentration between 7 and 15 μM (Figure 2B). These data revealed that EHX effectively inhibits the intra- and inter-cellular replication of CVB at a low concentration.

To further demonstrate the antiviral efficacy of EHX, we measured the viral RNA and protein level driven by the virus in the presence of EHX treatment. Briefly, HeLa cells were infected with CVB3 and were treated with twofold dilution of EHX, at the same time the virus infection began. In the virus control wells, only the culture medium was added. Total RNA and protein extracted at 24 hpi were analyzed using the RT-qPCR and Western blotting, respectively. As shown in Figure 2C, the viral RNA level progressively reduced over the range of concentration tested. EHX significantly reduced the level of viral RNA at 16 μM (*P* = 0.0001), 8 μM (*P* = 0.0037), and 4 μM (*P* = 0.0016) in treated cells. Similar results were observed for the Western blotting analysis that revealed that EHX significantly reduced the level of 3D protein at 32–8 μM (*P* < 0.0001), 4 μM (*P* = 0.0003), 2 μM (*P* = 0.0119), and 1 μM (*P* = 0.0104) when compared with the untreated cells. Likewise, compared with CVB3-infected cells without treatment, viral RNA, 3D protein, and virion production were significantly reduced in cells treated with favipiravir (Figure 2D).

Since EHX is presumably present at a lower concentration in its natural state, we further proceeded to evaluate the antiviral activity of EHX at lower concentration (i.e., nM). We found that at the lowest concentration (500 nM) tested in this study, EHX significantly inhibited the viral RNA (*P* = 0.0570) and 3D protein (*P* = 0.0166) level (Figure 2E). Taken together, EHX is a potent antiviral compound that effectively inhibits CVB replication *in vitro*.

### Ethyl 3-Hydroxyhexanoate Possesses Antiviral Activity Against Coxsackievirus B Infection *in vivo*

To demonstrate the antiviral activity of EHX against CVB infection *in vivo*, suckling Balb/c mice were infected with CVB3 intraperitoneally. The mice were administered 250 mg/kg (body weight) of EHX two times per day. Control mice were either treated with PBS or infected with CVB3. To determine the antiviral activity of EHX *in vivo*, mice were euthanized at day 5 pi, and the hearts were harvested for analysis.

**FIGURE 3:**
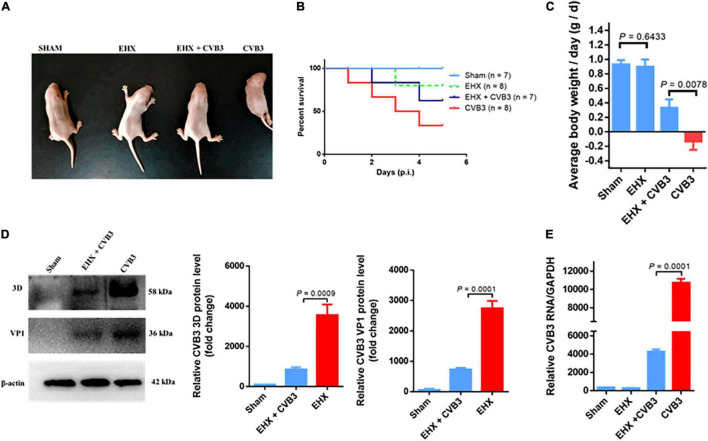
EHX inhibits CVB replication in virus-infected mice. Suckling mice (5 days old) were infected with CVB3 and were treated with EHX (250 mg/kg) two times a day for 5 days. **(A)** Representative picture of the overall physical condition of each mice group. **(B)** Survival rate of each mice group. **(C)** Average change in mouse bodyweight per day was calculated using the formula: (body weight-body weight of the previous day)/5 days. **(D,E)** EHX inhibits the viral protein and RNA level. Total protein and RNA were analyzed using Western blotting and RT-qPCR at the end of day 5 pi, respectively. Error bars represent SD, *n* = 3, Student’s *t*-test.

As shown in Figures 3A–C, CVB3 infection caused debilitating changes and loss of bodyweight in CVB3-infected mice group. Only about 37% (3/8) of the infected mice were alive at day 5 pi. In contrast, CVB3-infected mice group treated with EHX showed an improved survival rate as 57% (4/7) of the mice were alive at day 5 pi with improved general body appearance and gradual bodyweight gain during the course of the treatment. In addition, the level of viral 3D protein (*P* = 0.0009) and VP1 (*P* = 0.001) and RNA (*P* = 0.0001) were significantly reduced in the EHX-treated mice, indicating that EHX effectively inhibits CVB replication under *in vivo* scenario (Figures 3D,E).

### Viral Non-structural Proteins are the Potential Target of Ethyl 3-Hydroxyhexanoate

**FIGURE 4:**
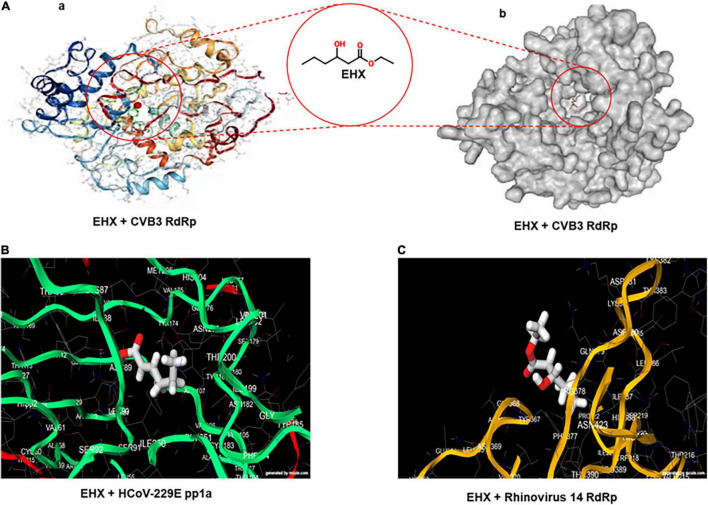
Viral non-structural protein is the potential target of EHX. **(A)** Three-dimensional structure of CVB RdRp showing the predicted binding site of EHX. The structures of the CVB RdRp were retrieved from the PDB (ascension number: 3DDK). Images were generated using the MedusaDock online software. **(B,C)** The predicted binding site of EHX with putative structure of human coronavirus-229E replicase polyprotein 1a and rhinovirus 14 RdRP. Images were generated on www.mcule.com/apps/1-click-docking/. HCoV-229E, human coronavirus-229E; pp1a, polyprotein 1a; RdRP, RNA-dependent RNA polymerase.

To gain a preliminary insight into the molecular mechanism of the antiviral activity of EHX, full virtual screening and molecular docking of EHX were carried out. Favipiravir was used as a standard. A docking score output of −5.0 was reported for the complex of favipiravir with poliovirus RdRp. EHX showed a high docking score against non-structural protein of seven different families and genera of RNA viruses, namely, *Enterovirus*, *Lentivirus*, *Arenavirus*, *Coronavirus*, *Flavivirus*, *Herpesvirus*, and *Orthomyxovirus*. The values of the binding energy were ranked and presented in Table 1. The docking score was used to determine protein–ligand interaction. Higher negative docking score represents a higher interaction between ligand and protein (high binding energy). Most of the non-structural proteins (ligand) binds with higher affinity with EHX. To confirm, the binding site(s) of EHX with CVB RdRp was predicted using the MedusaDock software. The dimeric structure of CVB3 RdRp (accession number: 3DDK) was retrieved from PDB. The result showed that EHX binds at acids THR57 and VAL51 (Figure 4) to CVB RdRp.

**TABLE 1:** The docking score of EHX with putative viral proteins.

Groups	Viruses	Proteins	Docking score	PDB ID
Coronavirus	Coronavirus 229E	Replicase 1a	−5.5	3EWR
Lentivirus	HIV 1 group M subtype B	Reverse transcriptase	−5.4	1DTQ
Herpesvirus	Herpes virus	Thymidine kinase	−5.2	1E2N
Arenavirus	Lassa virus	Nucleoprotein	−4.9	3MX2
Coronavirus	SARS coronavirus	Replicase 1a	−4.8	3MJ5
Flavivirus	Hepatitis C virus genotype 1b	RdRp	−4.6	3QGE
Enterovirus	Poliovirus 1	RdRp	−4.5	1RA7
Orthomyxo-viruse	Influenza virus	Neuraminidase enzyme	−4.4	1A4Q
Enterovirus	Rhinovirus 16	RdRp	−4.0	1TP7

*PDB, Protein Data Bank; VP1, Viral Protein 1; RdRp, RNA dependent RNA polymerase.*

### Ethyl 3-Hydroxyhexanoate Targets the Early Stage of Coxsackievirus Group B Replication

The replication of picornaviruses is a set of complex processes initiated by the binding of viral capsid protein with the host surface receptor. Penetration follows attachment. The process of attachment induces conformational changes in the viral capsid protein that results into receptor-mediated endocytosis. Alternatively, enterovirus can enter the host cells by capsid-receptor fusion. Following this is the virus uncoating process, aimed at delivering the viral genome to the cytosol for RNA transcription. The newly synthesis viral RNA serves as a template for virus protein translation (Thibaut et al., 2012). These steps are referred to as the early stage of virus replication.

**FIGURE 5:**
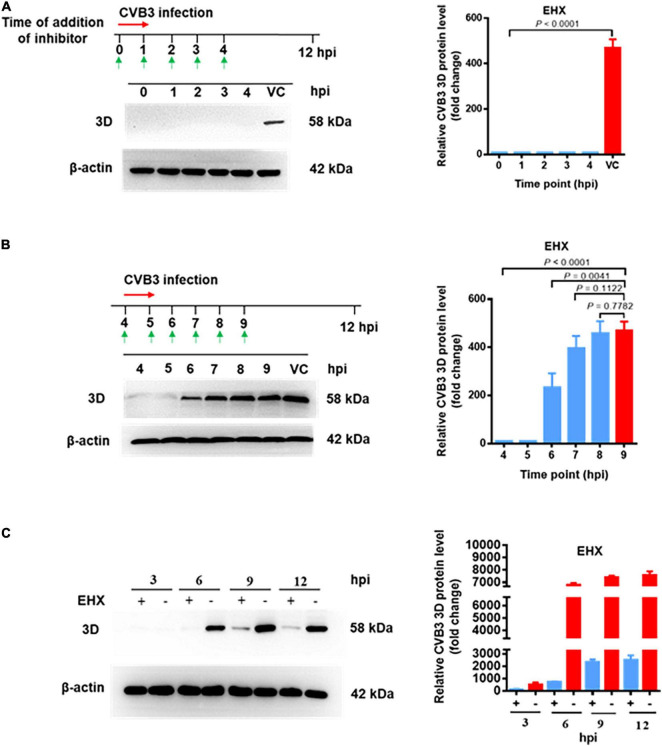
EHX inhibits CVB at the early stage of replication. **(A,B)** Time-of-addition assay. HeLa cells were infected with CVB3 (MOI = 1) and were treated with EHX (8 μM) at the indicated time points. Total protein was extracted and analyzed using Western blotting at 12 hpi. **(C)** CVB replication cycle. HeLa cells were infected with CVB3 (MOI = 1) and cultured in medium supplemented with EHX (8 μM). Total proteins were extracted at the indicated time points and analyzed using Western blotting.

To determine the step(s) at which EHX acts during CVB replication, a time-of-addition assay was carried out. In this assay, HeLa cells were infected with CVB3, and EHX (8 μM) was added to the culture medium at various time points of pi (illustrated in Figure 5A, upper diagram). Cell lysates were collected at 12 hpi and subjected to Western blotting. As shown in Figure 5A, the synthesis of viral protein was completely blocked when EHX was added up to 4 hpi.

To confirm the above observation, after CVB3 inoculation to HeLa cells, EHX was added to the culture medium at 4 hpi onward as indicated in Figure 5B. Total protein was extracted at the different time points, and the viral protein level was analyzed using Western blotting. The result showed that the synthesis of viral protein was blocked until 5 hpi. However, when the compound was added later than 6 hpi, the inhibitory effect of EHX gradually decreased. Above observations suggested that EHX inhibits CVB3 replication at the early stage of infection.

Motivated by the result of the time-of-addition assay, time course for the different early stage of events in CVB replication was roughly investigated. The virus replication cycle (without the addition of EHX) was examined in a parallel assay to determine the time point between virus transcription and translation. Briefly, HeLa cells were infected with CVB3 and were culture in medium with or without EHX. Cell lysate collected at different time point (interval of 3 h and up to 12 h of infection) were analyzed using Western blotting. As shown in Figure 5C, the synthesis of viral protein was completely not noticeable until the time point between 3 and 6 hpi; presumably, the transcription of viral RNA, which is the first intracellular step during CVB3 replication (Souii et al., 2013), has been initiated earlier before this time point. In culture medium supplemented with EHX, the synthesis of 3D protein was almost completely blocked until time point between 6 and 9 hpi. The data suggest that CVB RNA and protein synthesis occurred at the early stage of virus infection between 0 and 6 hpi *in vitro*. Taken together, we concluded that EHX exerts antiviral activity when it is applied at the early stage of CVB replication and that it could act at the step of viral RNA replication or protein synthesis.

### Ethyl 3-Hydroxyhexanoate Inhibits Coxsackievirus B Infection by Targeting Viral RNA Replication

**FIGURE 6:**
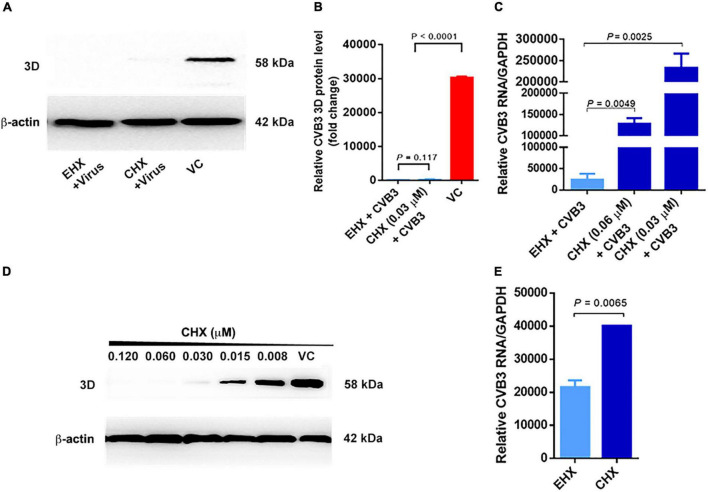
Viral RNA transcription step is the molecular target of EHX. **(A–C)** EHX and CHX significantly suppressed CVB3 RNA and 3D protein level. HeLa cells were infected with CVB3 (MOI = 1) and were treated with EHX (8 μM) and CHX (0.03 and 0.06 μM), respectively. Total protein and RNAs were extracted at 18 hpi and analyzed using Western blotting and RT-qPCR, respectively. **(D)** CHX minimum inhibitory concentration. HeLa cells were infected with CVB3 (MOI = 1) and were cultured in medium containing CHX at the indicated concentrations. Extracted total protein was analyzed using Western blotting at 18 hpi. **(E)** EHX reduced mini-CVB3 RNA. HeLa cells were transfected with mini-CVB3 (1 μg) and were treated with EHX (8 μM) and CHX (0.03 μM), respectively. Total RNAs were extracted 24 hpt and analyzed using RT-qPCR. Error bars represent SD, *n* = 3, Student’s *t-*test. CHX, cycloheximide; EGFP-C1, enhanced green fluorescent protein control 1.

To narrow down the early step(s) of virus replication blocked by EHX, cycloheximide (CHX) was used as a positive control to differentiate between translation and transcription. CHX is a known inhibitor of protein synthesis that binds to the E-site of the 60s ribosomal subunit, interfering with deacetylated tRNA of eukaryotic cells (Schneider-Poetsch et al., 2010). The quantification of viral protein and RNA expression in CVB3-infected cells were used to distinguish between the inhibition of viral RNA transcription and translation (Gao et al., 2015). To this end, CVB3-infected HeLa cells were cultured in a medium containing EHX (8 μM) and CHX (0.03 μM). Viral RNA and 3D protein were analyzed at 18 hpi. As shown in Figure 6A, the Western blotting analysis revealed that both EHX and CHX significantly reduced the level of viral 3D protein (*P* < 0.0001) when compared with the untreated cells. On one hand, there was no significant difference in the 3D protein (*P* = 0.117) level of the CVB3-infected cell treated with EHX, compared with CHX treatment. In contrast, the level of viral RNA (*P* = 0.0049) significantly reduced in CVB3-infected cells treated with EHX, compared with CHX (0.03 μM) treatment (Figures 6B,C,D). A similar result was obtained when the cells were treated with higher concentration of CHX (0.06 μM), suggesting that viral RNA synthesis significantly increased following CHX treatment but not in the presence of EHX.

Subsequently, we designed a CVB3 sub-genomic replicon, designated as “mini-CVB3.” The virus-based replicon contained the sequence of CVB3 P1-coding region flagged at both sides by CVB3’s 5′UTR and 3′UTR. This viral RNA is only capable of being transcribed, hence detectable by RT-qPCR. HeLa cells were transfected with the mini-CVB3 and cultured in a medium containing EHX and CHX, respectively. After 24 hpt, total RNAs were analyzed using RT-qPCR. As shown in Figure 6E, EHX treatment significantly reduced the mini-CVB3 RNA (*P* = 0.0065) level, as compared with CHX-treated cells.

Collectively, our data strongly suggest that EHX acts at the step of viral RNA transcription during CVB replication.

## Discussion

Enteroviruses are important human viruses. Each year, they affect millions of people worldwide (Bessaud and Delpeyroux, 2020). Of important is the poliovirus disease (Lugo and Krogstad, 2016). Other diseases caused by members of enteroviruses are hemorrhagic conjunctivitis, viral meningitis, and myocarditis. Given the high prevalence of enteroviruses in other mammalian species, it is plausible that enteroviruses that normally circulate in animal groups have a close relationship with and can infect humans (Prempeh et al., 2001; Lukashev and Vakulenko, 2017). While this is rare, there is a high risk of cross-species (animal to human) infection (Fieldhouse et al., 2018).

Coxsackievirus B is a possible cause of outbreak and has been implicated as a major etiologic agent of viral myocarditis, pericarditis, meningitis, and pancreatitis among children and young adults. More recently, CVB infection has been associated with the outbreak of hand, foot, and mouth disease (HFMD) (Han et al., 2019). While effort to develop effective anti-CVB drug is ongoing up to date, like most viral infections, there is no specific approved vaccine or treatment for CVB-related infection. Synthetic antiviral drug candidates with strong inhibitory activity against CVB and other enteroviruses have been identified, including fluoxetine (Zuo et al., 2012), Pleconaril (Fechner et al., 2011), and N-acetyl cysteine (Wang et al., 2020). In this study, we examined the antiviral activity of EHX and showed that it could elicit potent antiviral activity against CVB infection, a representative member of enteroviruses. EHX is a volatile compound in many fruits and vegetables. Extensive research has provided information about a wide range of natural products, and their use as antiviral agents (Lin et al., 2014).

Coxsackievirus group B preferentially replicates and induces a direct CPE in the infected cells (Tsueng et al., 2011). To establish the antiviral activity of EHX, we first demonstrated its ability to inhibit CVB-induced CPE. Our results revealed that EHX could effectively inhibit CVB-induced CPE with improved cell viability. Likewise, the result of our CPE-based dose-response analysis showed that both EHX and favipiravir (used as a positive control) inhibits CVB3 replication in a dose-dependent manner. EHX and favipiravir were found to have EC_50_ values of 1.2 and 62 μM, respectively, against CVB3. This finding is consistent with a previous study that reported favipiravir EC_50_ against Enterovirus-A71 (EV-A71) to be 68.74 μM (Wang et al., 2016). Favipiravir is active against severe acute respiratory syndrome coronavirus 2 (SARS-CoV-2) and Ebola virus, with an EC50 value of 61.88μM (Choy et al., 2020; Du and Chen, 2020). More importantly, with an EC_50_ value of 1.2 μM and an SI value of 20.8, EHX appears to be more potent than favipiravir. The high SI value suggests that EHX may be inhibiting a specific viral target (Chiang et al., 2005).

Our study further revealed that EHX significantly suppressed virus replication. Conventional virus quantification assay that was employed to quantify the amount of virus particles in CVB3-infected cells revealed that at concentration as low as 2 μM, EHX could inhibit virus replication up to twofold. Interestingly, the results from RT-qPCR and Western blotting also demonstrated that the EHX significantly reduced viral RNA and protein level at concentrations in nanomolar. Compounds that inhibit virus at lower concentrations have been proposed to have a good pharmacological safety profile (Mao et al., 2014). Likewise, EHX significantly reduced the virus titer at a relatively lower concentration than favipiravir, although the previous report revealed that favipiravir is a weak inhibitor (Wang et al., 2016). The difference in the antiviral activities of both compounds suggests the difference in the mechanism of their antiviral activity (Sun et al., 2015). While favipiravir is a nucleotide analog that was primarily designed to treat influenza virus infection (Furuta et al., 2017), EHX is a natural product that has no structural relationship with any nucleotide. We believe that this may contribute to the variation in the antiviral potency of EHX and favipiravir. Nonetheless, both compounds showed effective antiviral activity against CVB replication.

Based on the *in vitro* results, we investigated the antiviral activity of EHX *in vivo*. To this end, a murine model of CVB3-induced myocarditis (Burch et al., 1970, 1982; Huber, 2008) was treated with EHX and was observed if it would inhibit CVB replication and improve the overall condition of the mice. Interestingly, EHX treatment remarkably suppressed CVB3 replication and improved the overall condition and survival of virus-infected mice. Because EHX is a naturally occurring compound in fruits, food additives, and vegetables (Dunkel et al., 2009; Medina et al., 2020), the treatment of CVB-related infections with EHX can be well tolerated. Nonetheless, results from the animal model do not necessarily predict replication in human trials (Bracken, 2009); it is worthy of further investigations.

We sought to expand our evaluation of the antiviral activity and understand the mechanism of action of EHX; we started by virtually predicting the binding site(s) of EHX to viral proteins of different virus groups. Our findings revealed that EHX binds and interacts strongly to non-structural proteins of the viruses, with a rather high energy (Kitchen et al., 2004). Suggesting that it is possible it has more than one molecular target. The wide variety of viral targets of EHX possibly showed its broad diverse antiviral activities, as plant-based natural products are considered as reservoir and prolific source of chemicals with a very broad spectrum of biological activities (Pham et al., 2019; Mahavy et al., 2020). Furthermore, a study showed that plant-derived volatile compounds have short half-life and can be rapidly catabolized and degraded (Oikawa and Lerdau, 2013). However, our study demonstrated the antiviral effect of EHX at 24 h and up to 48 h after its addition to virus-infected cell culture. EHX’s antiviral ability to suppress CVB replication for a long period could be associated with its ability to bind tightly to viral protein components (ligands).

Motivated by the result of the virtual screening, we provided further insight into the mechanism of antiviral activity of EHX using different experimental methods. First, we performed a time-of-addition assay. According to the result, EHX exerts its antiviral activity between 0 and 5 h after virus infection. This suggested that its mode of antiviral action is at the early stage of virus replication. Events of early stage of CVB replication include binding of the virus to the receptor and internalization, subsequently followed by uncoating, translation, and transcription of viral genome (van der Linden et al., 2015; Li et al., 2020). To determine the specific virus replication event(s) targeted by EHX, CHX, a translation inhibitor (Santos et al., 2019), that disrupts translocation steps in protein synthesis, was used as a control to distinguish between viral protein and RNA synthesis inhibition (Schneider-Poetsch et al., 2010). CHX prevents translation of messenger RNA leading to an apparent decrease in protein synthesis and increase in the gene in question (Santos et al., 2019). Thus, we hypothesized that, compared with CHX, if EHX inhibits viral RNA replication, the treatment of virus-infected cells with EHX will result in a decrease in the viral 3D protein level with significant compensation (decrease) in the viral RNA level. To determine this, we quantified both viral protein and RNA abundance (Gingold and Pilpel, 2011). First, we showed that treatment of CVB3-infected cells with CHX and EHX at concentrations approximately between 0.03 and 0.06 μM and 8.0 and 16.0 μM could represent the minimum viral protein inhibitory concentration. At this concentration, no significance difference was observed in the 3D protein level of CVB3-infected cells treated with EHX, compared with CHX treatment. The viral RNA level of CVB3-infected cells treated with the same quantity of the compounds, however, differed significantly. In CHX-treated cells, viral RNA synthesis increased over time. This observation is likely due to a difference in the mechanism of antiviral activity between CHX and EHX, suggesting that EHX blocked the “accumulation” of viral RNA by inhibiting its transcription. Using the replicon inhibition assay, we further observed that following the EHX treatment, there was a significant reduction of the mini-CVB3 RNA level, compared with CHX treatment. Overall, our study suggested that EHX blocks viral RNA transcription during CVB infection.

The limitation of this study is that we have not demonstrated the direct binding of EHX with CVB 3D polymerase protein and determine the inhibition of its polymerase activity afterward. Although we believe that EHX targets CVB RNA transcription process as revealed by our data, this could be a starting point useful to unravel the detailed mechanism of the antiviral activity of EHX.

In conclusion, we demonstrated that EHX exerts potent antiviral activity against CVB infection *in vitro* and *in vivo*. Furthermore, based on the data presented in this study, we provided insight into the mechanism of the antiviral activity of EHX. We showed that it possibly acts at the step of viral RNA replication during CVB infection. EHX might be a safe therapeutic option and a promising strategy for the development of semi-synthetic novel antiviral drugs for viral diseases; therefore, it is worthy of future laboratory and clinical studies.

## Data Availability Statement

The raw data supporting the conclusions of this article will be made available by the authors, without undue reservation.

## Ethics Statement

The animal study was reviewed and approved by the Ethics Review Committee of Harbin Medical University, China.

## Author Contributions

OO performed the experiments and wrote the manuscript. JA, MY, and YC assisted in the animal care. JM and YF assisted in the collection of data. Z-HZ, WZ, and YW designed the study and reviewed the manuscript. The remaining authors provided substantial help for the implementation of this study. All authors contributed to the article and approved the submitted version.

## Conflict of Interest

The authors declare that the research was conducted in the absence of any commercial or financial relationships that could be construed as a potential conflict of interest.

## Publisher’s Note

All claims expressed in this article are solely those of the authors and do not necessarily represent those of their affiliated organizations, or those of the publisher, the editors and the reviewers. Any product that may be evaluated in this article, or claim that may be made by its manufacturer, is not guaranteed or endorsed by the publisher.

## Footnotes

1
www.mcule.com/apps/1-click-docking
